# Primary Hyperparathyroidism Secondary to a Sestamibi-Negative Cystic Parathyroid Adenoma

**DOI:** 10.7759/cureus.17577

**Published:** 2021-08-30

**Authors:** George M Win, Timur Gusov, FNU Marium, Michael J Gardner

**Affiliations:** 1 Diabetes and Endocrinology, University of Missouri, Columbia, USA; 2 Internal Medicine, University of Missouri, Columbia, USA

**Keywords:** hyperparathyroidism treatment, parathyroid gland adenoma, parathyroid cyst, hypercalcemia, tc99m sestamibi scan

## Abstract

The most common cause of primary hyperparathyroidism (PHPT) is a solid parathyroid adenoma. Less than 2% of cases of PHPT are caused by cystic parathyroid adenomas formed from degeneration of pre-existing solid parathyroid adenomas. Cystic parathyroid adenomas are non-functional in over 90% of cases. In this case we describe management of a 56-year-old man with acute-onset polydipsia, polyuria, xerostomia, nausea, and constipation. Serum chemistry upon admission revealed hypercalcemia, hyperparathyroidism, and reduced serum phosphorus. Neck sonogram revealed a predominantly anechoic lesion later confirmed by pathology to be a cystic parathyroid adenoma in the right thyroid lobe. Sestamibi scan did not show uptake in parathyroid gland, and parathyroid hormone (PTH) was elevated in fine-needle aspiration sample. Otolaryngology removed the cystic lesion via surgical excision, which led to normalization of PTH level. This case demonstrates the importance of evaluation of cystic components for PTH levels and if confirmed should be treated as a parathyroid adenoma.

## Introduction

Primary hyperparathyroidism (PHPT) refers to excessive secretion of parathyroid hormone (PTH) from the parathyroid glands. PHPT is associated with complications such as nephrolithiasis, osteoporosis, and neurocognitive defects. It can be acquired or inherited as in hereditary endocrine syndromes such as multiple endocrine neoplasias, hereditary hyperparathyroidism-jaw tumor syndrome, and familial isolated hyperparathyroidism [1]. The most common cause of PHPT is from a solid parathyroid adenoma [2]. Cystic parathyroid adenomas are believed to be the cause of less than 2% of cases of PHPT [3]. Cystic parathyroid adenomas are thought to be produced from cystic degeneration of previously existing solid parathyroid adenomas [4]. Other proposed etiologies including retention of glandular secretions and persistence of pharyngeal pouches have been proposed. Cystic parathyroid adenomas are non-functional in over 90% of cases and are reported to be more common in males [2]. We describe a case of PHPT secondary to a 99mTc sestamibi-negative parathyroid cystic adenoma in a patient with presentation of acute-onset polydipsia, polyuria, xerostomia, nausea, and constipation.

## Case presentation

Our patient is a 56-year-old man with history of hepatitis C and alcohol misuse disorder. He was transferred to University Hospital (UH) from Boone Hospital with nausea, vomiting, and concerns of hypercalcemia. He drinks 6-8 beers/day and shots of whiskey every day. He quit smoking two years ago but reports a history of smoking one pack of cigarettes per day for 35 years. He denied dysuria, fevers, headache, chest pain, and cough. He was in a state of usual health until about seven days prior when he began feeling worsening fatigue, weakness, polyuria, and polydipsia. He was seen in an outside hospital (OSH) emergency room two days earlier for his worsening fatigue, where he was told he has hypercalcemia and elevated blood pressure. Workup at OSH revealed elevated Ca^2+^ (15.8 mg/dL), hypokalemia (3.2 mmol/L), and normal serum creatinine (1.2 mg/dL). Patient was treated with IV fluids, 40 mEq KCl, and lorazepam. Patient was discharged home the same day. He reports that he did not notice any improvement and symptoms persisted the next day. He had borderline enlarged submandibular lymphadenopathy and blood pressure 195/105. He endorsed several episodes of vomiting, nausea, xerostomia, and dull bilateral lower back pain. Additionally, constipation was noted for one week. He decided to go to Boone Hospital because of his symptoms.

Upon admission to UH, physical examination was normal. Patient was afebrile with blood pressure of 170/120 mmHg. Serum chemistry after admission to UH showed hypercalcemia (14.5 mg/dL), hypophosphatemia (2.2 mg/dL), elevated intact PTH (375 pg/mL), low 25-OH vitamin D (19 ng/mL), and elevated serum creatinine (1.22 mg/dL). On days 1-3 of admission, an endocrinologist was consulted and he was started on aggressive hydration with normal saline (NS)/lactated ringer, IV potassium phosphate 15 mmol in 250 of NS, 2 packets (8 mmol phosphorous and 7 mEq of potassium) TID, IV calcitonin 350 units once, and zoledronic acid 4 mg IV once.

**Figure 1:**
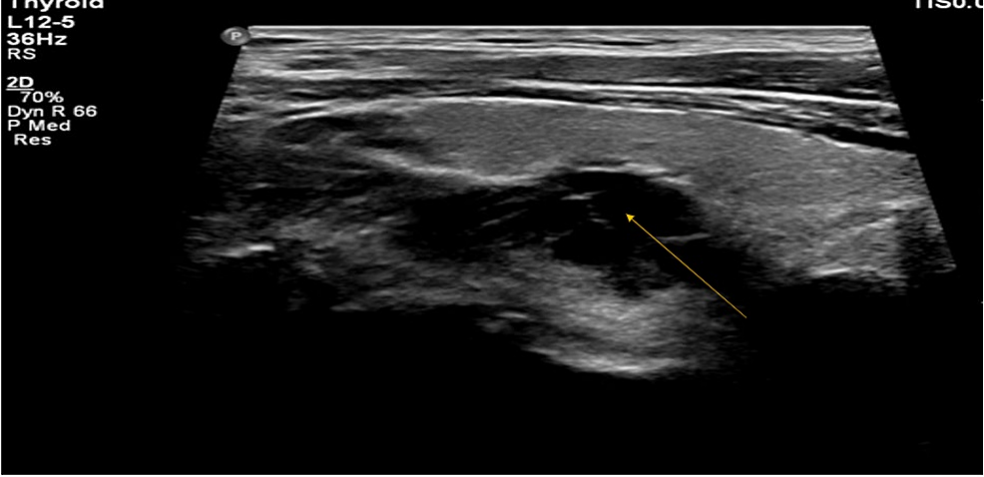
Thyroid Lobe (R) Ultrasound Neck sonogram highlighting anechoic lesion with septa in the right thyroid lobe

Neck sonogram revealed a large complex predominantly anechoic lesion with solid vascular components and thick internal septations in the inferior and medial aspect of the right thyroid lobe with maximum size 3 x 2 x 5.5 cm (Figure 1). Composition was mixed cystic and solid, with an incidentally noted heterogeneous hypodense nodule arising from the posterior right lobe of the thyroid with posterior peripheral hyperdensities, measuring 2.7 x 2.0 x 4.0 cm in the anteroposterior (AP), right/left (RL), and superoinferior (SI) dimensions.

**Figure 2:**
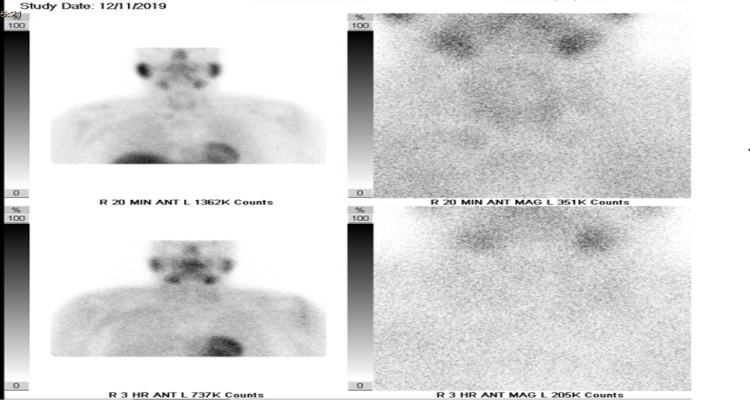
Tc-99m Sestamibi Impression: No evidence of parathyroid adenoma. There is focal area of increased sestamibi uptake in the posterior portion of left lung lower lobe. No findings on CT images (lung window) at this location. This finding likely represents a radioactive sestamibi embolus during IV injection of sestamibi.

**Figure 3:**
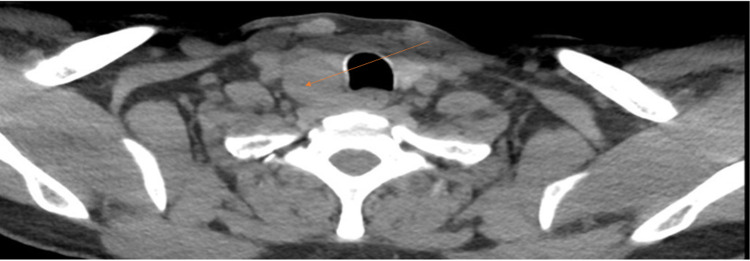
Chest CT on Day 5 of Admission Impression: Large predominantly cystic lesion with tiny enhancing internal nodular components lying on the external aspect of the inferior right lobe of the thyroid most likely representing a cystic parathyroid adenoma

Parathyroid scan (planar and single-photon emission computed tomography/CT) for concern of hyperparathyroidism was completed using Tc-99m sestamibi in the thyroid bed and mediastinal region. No focal or increased radiotracer uptake was noted and impression noted no evidence of parathyroid adenoma (Figure 2). CT of the chest on day 5 revealed a large predominantly cystic lesion with tiny enhancing internal nodular components external to the inferior right lobe of the thyroid most likely representing a cystic parathyroid adenoma which was likely negative on nuclear medicine scan because of its cystic component (Figure 3).

**Figure 4:**
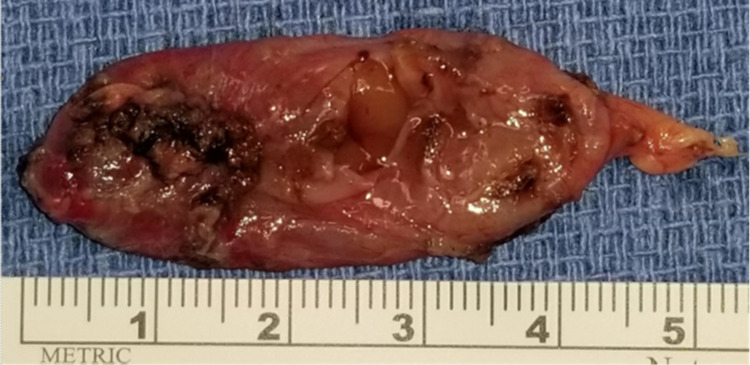
Surgical Pathology Specimen Intraoperative section analysis revealing cystic parathyroid tissue favoring parathyroid adenoma with focal atypia

On day 7 of hospital admission, the patient became irritated with medical personnel and reported that he was tired to be in the hospital. He asked to be discharged and received recommendations of oral hydration, endocrinology and otolaryngology follow-up as soon as possible. Approximately one month after discharge, patient underwent fine-needle aspiration (FNA), which was unable to detect cellular material but PTH was >100 pg/mL. Approximately one month after FNA, otolaryngology services performed a right upper parathyroidectomy with no changes in serum PTH detected. The cystic lesion was removed next with normalization of serum PTH (from 216 to 35.2 pg/mL) (Figure 4). Intraoperative section analysis revealed cystic parathyroid tissue favoring parathyroid adenoma with focal atypia. The patient’s hypercalcemia resolved.

## Discussion

The true prevalence of parathyroid cysts regardless of functionality is unknown among the general population, with estimates ranging from 0.075% to 11% [5]. The largest retrospective study examining the actual prevalence of cystic parathyroid adenomas occurred at Peking University Medical College and found a prevalence of 4.1% in 907 patients who underwent parathyroidectomy. Although a rare cause of PHPT, patients with cystic parathyroid adenomas are more likely to present with hypercalcemic crisis compared to solid adenomas.

PHPT secondary to functional cystic parathyroid adenomas can pose challenges to clinicians, not only because they are rare, but also as negative sestamibi scans may erroneously increase suspicion for a thyroid etiology of functional parathyroid cysts. One case of a cystic parathyroid adenoma was initially diagnosed as a cystic nodular goiter and inconclusive PHPT as the patient declined FNA [6]. There are multiple treatment options for cystic parathyroid adenomas. For instance, ultrasound-guided FNA resulting in collapse of cystic parathyroid adenomas has been reported. Additionally, aspiration of cystic parathyroid adenomas has been reported with a relatively low success rate [5]. Medical management with palmidronate and calcitonin was unable to resolve manifestations of a functional cystic parathyroid adenoma in another case. This patient’s hypercalcemic crisis was later successfully resolved after surgical excision of a cystic parathyroid adenoma [7]. Intracystic sclerosing therapy to resolve functional cystic parathyroid adenomas with tetracycline or ethanol has also been proposed; however, this method has a higher risk of nerve damage and morbidity compared to surgical alternatives. Overall, surgical excision of cystic parathyroid adenomas has an excellent prognosis and is considered first-line treatment in management of functional cystic parathyroid adenomas [8].

## Conclusions

PHPT secondary to a cystic parathyroid adenoma is very rare, as less than 10% of parathyroid cysts are functional. This case report highlights a case of a patient who presented with hypercalcemic crisis with negative 99mTc sestamibi scan. In patients presenting with hyperparathyroidism with a cystic component, the cystic component should be evaluated for PTH levels and may warrant treatment surgical excision as a parathyroid adenoma. 
